# Surface Porousization of Hard Carbon Anode Materials for Sodium-Ion Batteries

**DOI:** 10.3390/mi16070771

**Published:** 2025-06-30

**Authors:** Qianhui Huang, Shunzhang You, Chenghao Yang

**Affiliations:** 1 China Southern Power Grid Technology Co., Ltd., Guangzhou 510080, China; keelhuang1116@gmail.com; 2 Guangzhou Key Laboratory for Surface Chemistry of Energy Materials, New Energy Research Institute, School of Environment and Energy, South China University of Technology, Guangzhou 510006, China; 202210189203@mail.scut.edu.cn

**Keywords:** sodium-ion batteries, hard carbon, electrochemical performance, specific capacity, initial coulombic efficiency

## Abstract

Sodium-ion batteries (SIBs) have been considered as a promising alternative to lithium-ion batteries (LIBs) for large-scale energy storage. However, the commercial graphite anode is not suitable for SIBs due to its low Na^+^ ion storage capability. Currently, hard carbon has been considered a promising anode material for SIBs. Herein, the surface porousized hard carbon anode materials have been prepared by using hydrogen peroxide (H_2_O_2_) with a hydrothermal method (HC-HO) and utilized as the anode material for SIBs. The porous structure of HC-HO provides more storage space for Na^+^ ions and enhances the intercalation/deintercalation reversibility and diffusion rate of Na^+^ ions. Moreover, HC-HO can effectively alleviate the particle volume expansion and generate a thin and stable SEI film during charge/discharge processes. Thus, the HC-HO exhibits a high reversible capacity (314.4 mAh g^−1^ with an ICE of 92.3% at 0.05 C), excellent rate performance (241.4 mAh g^−1^ at 3 C), and outstanding cycling stability (a capacity retention of 78.6% after 500 cycles at 1 C). The preparation of porous hard carbon provides new ideas for the future development direction of hard carbon.

## 1. Introduction

Recently, the increasing dependence on fossil fuels has highlighted worldwide energy and environmental concerns. This has led to the fast-growing utilization of renewable energy sources, including wind and solar power [1,2,3]. However, the randomness and discontinuity characteristics of these natural energy resources make them challenging in practical applications. Thus, reliable and stable energy storage technology is urgently needed to promote the applications of these renewable energies [4,5]. Currently, sodium-ion batteries (SIBs) have attracted much attention due to their excellent electrochemical performance at low temperature and high safety features and the natural abundance of sodium resources [6,7]. Although the working mechanism of SIB is similar to that of lithium-ion batteries (LIBs), the traditional graphite anode is not suitable for Na^+^ ions storage [8,9]. Therefore, great efforts have been focused on developing suitable anode materials for SIBs, e.g., hard carbon [10,11], soft carbon [12,13], and graphene [14,15]. Other carbonaceous materials [16,17] have been fabricated and investigated. Among them, hard carbon shows a high reversible specific capacity of over 300 mAh g^−1^ and a low operation voltage of 0.1 V, which make it the most promising anode material for SIBs [18,19].

Hard carbon consists of many randomly arranged layers and small, curved graphene nanosheets, forming a large granular particle with a large number of defects and nanopores inside it [20,21]. Hard carbon can be prepared from biomass carbon sources or polymer materials for their wide availability and environmental friendliness [22,23]. However, the commercialization of these fabricated hard carbon materials is constrained by a low initial coulombic efficiency (ICE) and poor rate performance and cycling stability [24,25,26]. To promote the practical applications of hard carbon in SIBs, researchers are committed to studying their performance optimization strategies. In recent years, lots of optimization methods have been studied, which can be classified as follows, including structural regulation [27,28], morphological design [29,30], interfacial construction [31,32], and electrolyte optimization [33,34,35,36]. Structural regulation has been considered as the most effective way to improve the electrochemical performance of carbonaceous-based SIB anode materials. For example, Yu et al. synthesized three-dimensional amorphous carbon with controllable porous and disordered structure by a simple NaCl template-assisted method [37]. Hu et al. prepared hard carbon with a closed pore structure by using ethanol as the pore-forming agent and phenol–formaldehyde resin as the carbon source with a Na^+^ storage capacity up to 410 mAh g^−1^ [38]. Liu et al. obtained hard carbon with low defects and a high ICE of 86.1% by regulating the heating rate during the fabrication process [39].

**Figure 1:**
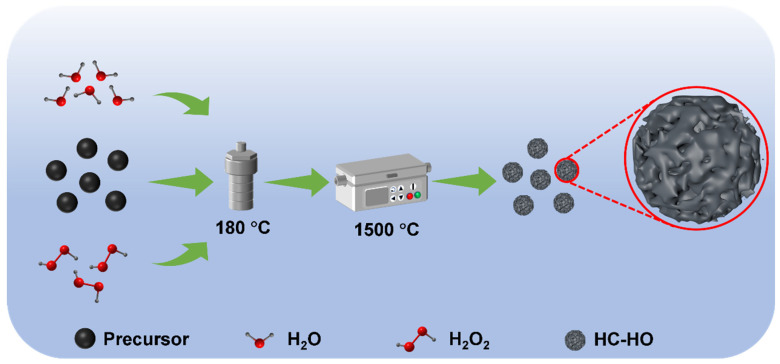
Schematic illustration for the fabrication of porous hard carbon by using hydrogen peroxide (H_2_O_2_) with a hydrothermal method (HC-HO).

Herein, the surface porousized hard carbon materials have been prepared by using hydrogen peroxide (H_2_O_2_) with a hydrothermal method (HC-HO). During the high-temperature carbonization process, H_2_O_2_ is decomposed to produce water vapor and oxygen at high temperature. The generated gases can create pores in the hard carbon precursor particles, and the HC-HO was successfully obtained (Figure 1). The larger specific surface area of HC-HO provides more space for Na^+^ ion adsorption, and the increased number of pores can provide more paths for Na^+^ ion migration. In addition, the reversible extraction/insertion of Na^+^ ions can alleviate the volume expansion of hard carbon particles as well as the generation of solid electrolyte interface (SEI) film, effectively reducing the interfacial resistance. Thanks to the advantage of a unique structure, HC-HO achieves a high reversible capacity of 314.4 mAh g^−1^ at 0.05 C (1 C = 300 mA g^−1^) with a high ICE of 92.3%. The fabricated HC-HO also shows a high rate performance and cycling stability, and it can reach 226.1 mAh g^−1^ with the capacity retention of 78.6% at 1.0 C after 500 cycles.

## 2. Experimental Section

### 2.1. Materials Fabrication

Hard carbon precursors were obtained from Guangdong Rongna New Energy Technology Co., Ltd., Guangzhou, China. The 1 M NaCF_3_SO_3_ (NaOTF)–diethylene glycol dimethyl ether (DEGDME) electrolyte was purchased from DoDoChem, Suzhou, China. The Na metal (>99.7%) and hydrogen peroxide (H_2_O_2_, AR, 30 wt% in H_2_O) were purchased from Aladdin, Shanghai, China.

### 2.2. Preparation of HC-HO

The 5 g hard carbon precursor, 15 mL H_2_O_2_, and 60 mL H_2_O were mixed and stirred for 2 h to obtain a disperse solution. The as-obtained disperse solution was transferred into a 100 mL Teflon-lined autoclave and heated at 180 °C for 6 h. The product after hydrothermal treatment is washed with deionized water three times and dried at 80 °C for 12 h. Finally, the dried product was carbonized at 1500 °C for 2 h to obtain HC-HO under a N_2_ atmosphere. The hard carbon anode materials fabricated without adding H_2_O_2_ (HC) are studied for comparison.

### 2.3. Material Characterizations

Crystal structures of HC and HC-HO samples were characterized by using X-ray powder diffraction (XRD, Bruker D8, Germany, Cu Kα, λ = 1.5418 Å). Nitrogen adsorption/desorption isotherm measurements and pore analysis were conducted at 77 K using an Autosorb iQ apparatus. Raman spectra of the HC and HC-HO samples were performed by using a Raman spectrophotometer (523 nm Ar laser, LabRAM HR Evolution, Horiba, Tianjin, China). The morphology of HC and HC-HO samples was observed by field-emission scanning electron microscopy (FESEM, Hitachi SU8010, Hitachi, Tokyo, Japan) and transmission electron microscopy (TEM, JEM-2100Plus, JEOL, Tokyo, Japan).

### 2.4. Electrochemical Measurements

HC-HO or HC was mixed with super P and carboxyl methyl cellulose (CMC) with a mass ratio of 96:2:2 in distilled water to obtain a slurry. The slurry was coated on copper foil with a thickness of 150 μm and dried at 80 °C overnight. Then, the dried electrode was sliced into a 13 mm disc. The mass loading of HC-HO or HC electrode is about 3–4 mg cm^−2^. The 2032 coin-cells were assembled using Na foil (the diameter is 16 mm) as the counter electrode, 1 M NaOTF-DEGDME as the electrolyte (150 μL), and glass fiber as the separator (the diameter is 19 mm). Charge/discharge tests were performed on a LAND CT2001 battery testing system at 25 °C with a voltage range of 0.01–2.5 V. EIS and CV tests were performed on a CHI660e electrochemical workstation. The parameters of EIS were set with a frequency range from 100 kHz to 0.01 Hz with an AC amplitude of 0.005 V. The parameters of GITT were set with a current pulse duration of 20 min at 0.1 C and an interval time of 3 h.

The coefficient diffusion of Na^+^ ions measured from CV is calculated as follows:

$$I_{p}=\left(2.69\times 10^{5}\right)n^{3/2}AD^{1/2}Cv^{1/2}$$

where $I_{p}$ is the peak current (A), n is the number of reaction electrons, A is the contact area between the electrolyte and electrode (cm^2^), D is the diffusion coefficient Na^+^ ions (cm^2^ s^−1^), C is the concentration of Na^+^ ions in the electrode (mol cm^−3^), and v is the scan rate (V s^−1^).

## 3. Results and Discussion

**Figure 2:**
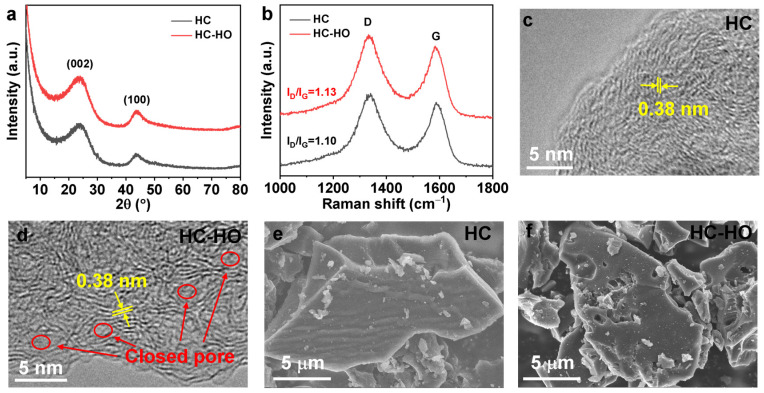
XRD patterns (**a**) and Raman spectroscopy (**b**) of HC and HC-HO. HR-TEM (**c**,**d**) and SEM (**e**,**f**) images of HC (**c**,**e**) and HC-HO (**d**,**f**), respectively.

X-ray diffraction (XRD) patterns of fabricated HC-HO and hard carbon without H_2_O_2_ (HC) are shown in Figure 2a. As indicated in the figure, the X-ray diffraction (XRD) patterns of HC-HO and HC exhibit two broad peaks at 23.9 and 43.7°, corresponding to the lattice planes of (002) and (100), respectively. The (002) and (100) planes of HC-HO do not shift, suggesting that the gases produced by H_2_O_2_ do not change the interlayer spacing of HC. Figure 2b displays the Raman spectroscopy of HC and HC-HO. The D and G peaks located at 1354 and 1600 cm^−1^ can be observed in the Raman spectra, respectively. The D band originates from defective graphite structure or disordered carbon atoms at the graphite sheet edges. Meanwhile, the G band is related to the in-plane C–C bond stretching of sp^2^-hybridized graphitic carbon atoms. They represent the induced band and crystalline graphite band in the graphite sheet, respectively. The intensity ratios of the D and G bands (I_D_/I_G_) can be used to analyze the degree of disorder in HC-HO and HC. As shown in the figure, the I_D_/I_G_ of HC-HO is 1.13, which is larger than that of HC (1.10), indicating that the gases produced by H_2_O_2_ will induce some defects and enhance the disorder degree of carbon atoms in HC-HO. The interlayer spacing of both HC and HC-HO can be observed and measured by a high-resolution transmission electron microscope (HR-TEM) to be 0.38 nm (Figure 2c,d), which is consistent with the XRD results. Moreover, from the HR-TEM shown in the figures, it is detected that the carbon layer structure of HC-HO is more disordered than that of HC. The morphologies of HC and HC-HO were studied by using a scanning electron microscope (SEM). The surface of HC particles was relatively smooth (Figure 2e), while obvious pores with different sizes were observed on the HC-HO particles’ surface (Figure 2f), indicating that H_2_O_2_ does play an effective role in pore formation on its surface. The specific surface areas of HC and HC-HO were measured by utilizing nitrogen adsorption/desorption isotherms. As shown in Figure S1a, the specific surface area of HC-HO is 78.27 m^2^ g^−1^, which is much higher than that of HC (11.99 m^2^ g^−1^). The pore size distribution showed that HC-HO had pores between as many as 3 and 10 nm (Figure S1b), and TEM also proved that there were closed pore structures in HC-HO, which can provide sufficient space for the storage of Na^+^ ions.

**Figure 3:**
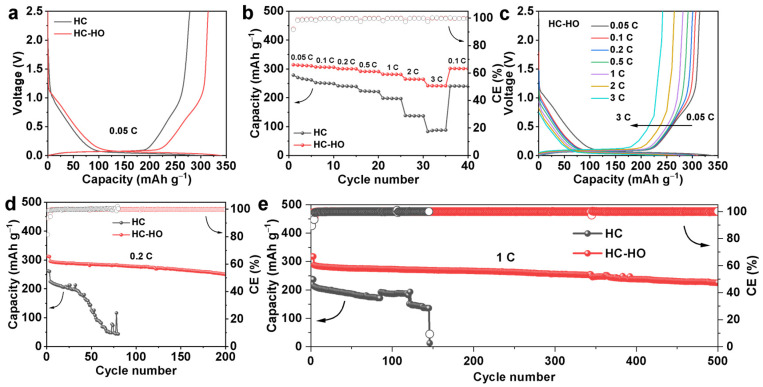
The galvanostatic charge/discharge (GCD) curves of the first cycle at 0.05 C (**a**) and rate performance (**b**) of HC and HC-HO. The charge/discharge profiles at different current densities of HC-HO (**c**). Cycling performance of HC and HC-HO at 0.2 C (**d**) and 1.0 C (**e**).

To verify the effectiveness of the porous structure, electrochemical properties of HC and HC-HO as SIB anodes have been investigated by using 2032 coin-type half cells, with Na metal foils as the counter and reference electrode. As indicated in Figure 3a, HC-HO can reach a reversible capacity of 314.4 mAh g^−1^ in the first cycle with an ICE of 92.3%, which is better than HC for 278.5 mAh g^−1^ with an ICE of 91.6%. Moreover, the galvanostatic charge/discharge (GCD) curves reveal a significant enhancement of both the slope and plateau capacities of HC-HO. As shown in Figure S2, the slope discharge capacity is enhanced from 91.1 mAh g^−1^ for HC to 112.5 mAh g^−1^ for HC-HO, while the plateau capacity is increased from 212.7 mAh g^−1^ for HC to 228.3 mAh g^−1^ for HC-HO. It confirms that the porous structure in HC-HO promotes the adsorption and filling of Na^+^ ions’ pores and defects. In addition, the porous structure of HC-HO promotes the diffusion of Na^+^ ions and improves the rate performance. HC-HO can reach a reversible capacity of 312.1, 306.1, 300.4, 291.3, 281.3, 264.5, and 241.4 mAh g^−1^ at the current densities of 0.05 C, 0.1 C, 0.2 C, 0.5 C, 1 C, 2 C, and 3 C (1 C = 300 mA g^−1^), respectively. The reversible capacity reaches 301.2 mAh g^−1^ when the current density goes back to 0.1 C (Figure 3b,c). In contrast, the reversible capacity of HC can only reach to 266.3, 250.5, 240.2, 222.4, 197.6, 137.1, and 87.8 mAh g^−1^ at the current densities of 0.05 C, 0.1 C, 0.2 C, 0.5 C, 1 C, 2 C, and 3 C, respectively (Figure S3). When the current rate is set back to 0.1 C, it can only deliver a capacity of 240.6 mAh g^−1^. The porous structure of HC-HO not only enhances the Na^+^ storage capacity and rate performance, but also improves its cycle stability significantly. HC-HO exhibits a capacity retention of 84.7% at 0.2 C after 200 cycles (Figure 3d and Figure S4a). Moreover, HC-HO can reach a high reversible capacity of 226.1 mAh g^−1^ at 1.0 C with a capacity retention of 78.6% after 500 cycles (Figure 3e and Figure S4b). However, HC shows obvious decay after 50 cycles under the current density of 0.2 C, and the battery could not work normally after 150 cycles at the current density of 1.0 C (Figure 3d,e and Figure S5). The main reason is that HC has poor Na^+^ ion storage reversibility in HC, generating more by-products and dead sodium, leading to the rapid decay of the battery capacity or even structure collapse. It benefits from a porous structure and enriched defects on the pore surface in HC-HO and exhibits a high reversible capacity and ICE surpassing most of the hard carbon materials as reported elsewhere (Table 1 and Figure S6). We also use NaNi_1/3_Fe_1/3_Mn_1/3_O_2_ (NFM) as the cathode and assemble it into a full cell for testing. As shown in Figure S7, the NFM//HC-HO can cycle stably at 1 A.

**Figure 4:**
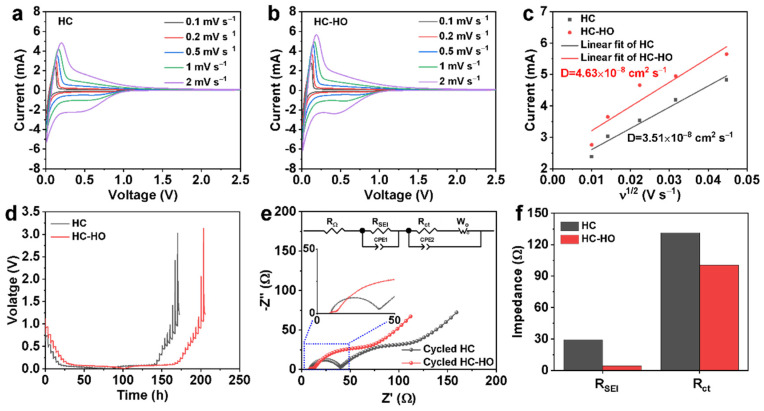
CV curves at different scan rates (**a**,**b**), the relationship between the peak current (I_p_) and the square root over scan rate (ν^1/2^) (**c**), GITT curves (**d**), HC (**a**,**c**,**d**), and HC-HO (**b**–**d**), respectively. The EIS spectra (**e**) and the corresponding R_SEI_ and R_ct_ (**f**) of HC and HC-HO anode after 200 cycles at 0.2 C. The insets of (**e**) present a high-magnification graph and equivalent circuit.

To compare the kinetic behavior of HC and HC-HO, the 2032 coin-type half cells assembled with HC or HC-HO as anode and Na metal as the counter electrode were subjected to cyclic voltammetry (CV) tests at different scan rates (0.1, 0.2, 0.5, 1, 2 mV s^−1^). The CV curves of HC and HC-HO at the voltage range of 0.01–2.5 V are shown in Figure 4a,b. The peak currents of HC-HO are much higher than those of HC at each scan rate, indicating the faster diffusion of Na^+^ ions in HC-HO. By fitting the peak currents at different scan speeds, the diffusion coefficient of Na^+^ ions (D_Na+_) in HC-HO can be calculated to be 4.63 × 10^−8^ cm^−2^ s^−1^, which is much higher than the D_Na+_ in HC (3.51 × 10^−8^ cm^−2^ s^−1^) (Figure 4c). The galvanostatic intermittent titration technique (GITT) testing results can also show that the D_Na+_ in HC-HO is greater than that of HC (Figure 4d). D_Na+_ is one of the main parameters that determine their rate performance; thus, the rate performance of HC-HO is better than HC. In addition, electrochemical impedance spectroscopy (EIS) tests were performed on HC and HC-HO before and after cycling. As shown in Figures S8 and S9, and Figure 4e, a semicircle in the high-frequency region and a straight line in the low-frequency region can be observed in these EIS profiles. The depressed semicircle in the high-frequency range corresponds to the charge-transfer resistance (R_ct_) in the HC or HC-HO anode–electrolyte interface. Meanwhile, the intercept on the Zreal axis corresponds to the ohmic resistance (R_Ω_) coming from the electrolyte. Simplified equivalent circuit models (Figure S8a and Figure 4e) were constructed to study the impedance spectra of HC and HC-HO before (Figure S8b) and after cycling at 0.2 C for 200 cycles (Figure 4e). As exhibited in Tables S1–S3, the R_ct_ of HC-HO is much lower than that of HC, indicating that the porous structure also benefits charge transfer. Meanwhile, the resistance originating from the SEI film (R_SEI_) and R_ct_ of HC-HO after cycling are much lower than those of HC (Figure 4f and Figure S9b, Tables S2 and S3), indicating that the porous structure of the hard carbon is able to alleviate the continuous generation of SEI during cycling and form a stable interface, which is conducive to the transfer of electrons and Na^+^ ions.

**Figure 5:**
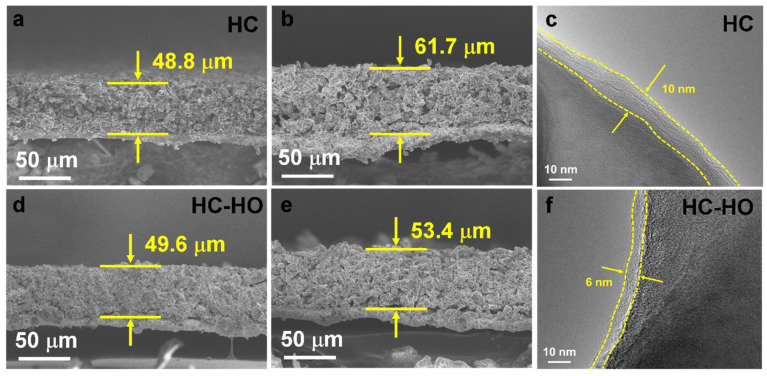
Cross-sectional SEM of HC (**a**,**b**) and HC-HO (**d**,**e**) anodes before and after 200 cycles at 0.2 C. TEM of (**c**) HC and (**f**) HC-HO anodes after 200 cycles at 0.2 C.

The HC and HC-HO hard carbon anodes at 0.2 C after 200 cycles have been further analyzed for changes in volume and interface. By comparing the cross-sectional SEM of the HC and HC-HO electrodes before and after cycling, the volume of the HC electrode increases from 48.8 to 61.7 μm with a thickness expansion rate of 1.26 (Figure 5a,b). Meanwhile, HC-HO expands from 49.6 to 53.4 μm after cycling with a volume expansion rate of only 1.07 (Figure 5d,e). The reversible intercalation/deintercalation of Na^+^ ions in the porous structure of HC-HO greatly alleviates the volume expansion of hard carbon particles. A larger volume expansion can cause the peeling off of the HC anode materials during the cycling process, resulting in a rapid decline of the specific capacity. In addition, it can be observed through TEM that a SEI film of approximately 10 nm is formed on the surface of HC after cycling (Figure 5c), while it is only 6 nm for HC-HO (Figure 5f). This also confirms that HC consumes more Na^+^ ions and generates an unstable SEI, leading to the capacity decay [47,48]. A uniform and stable SEI can promote electron transfer and maintain robust structural stability, which is consistent with the results of EIS. The porous structure of HC-HO not only effectively alleviates the volume expansion of hard carbon particles during the charging and discharging process, but also reduces the degradation of specific capacity, thus enhancing the Na^+^ storage capacity and cycling stability. As shown in Figure S10a,c, the C 1s peaks can be divided into C-C/C-H, C-O, C=O, C-F, and -CF_3_. For the O 1s spectrum in Figure S10b,d, the three peaks can be assigned to Na-O/C=O, O-C/O-H, and Na-KLL. Among them, the surface of HC after cycling is significantly enriched with more sodium compounds, leading to an increase in EIS.

## 4. Conclusions

Surface porousized hard carbon anode materials have been prepared by using hydrogen peroxide (H_2_O_2_) with a hydrothermal method (HC-HO). During the fabrication process, the released gas from hydrogen peroxide under high temperature and pressure can effectively improve the porosity of the hard carbon anode materials. The abundant porous structure on the HC-HO surface can not only provide additional storage sites for Na^+^ ions, but also facilitate the rapid diffusion of Na^+^ ions. Furthermore, HC-HO can effectively alleviate the particle volume expansion and generate a thin and stable SEI film during charge and discharge processes. As a result, HC-HO can achieve a reversible capacity of 314.4 mAh g^−1^ in the initial cycle with a high ICE of 92.3%. Compared to pristine HC, the rate performance and cycling stability of HC-HO have also been significantly improved. HC-HO exhibits an enhanced cycle stably for 200 cycles at 0.2 C and 500 cycles at 1.0 C with capacity retention rates of 84.7% and 78.6%, respectively. The preparation of this porous hard carbon provides a new idea for the development of hard carbon anode materials.

## Figures and Tables

**Table 1:** Comparisons of electrochemical performance for HC anodes.

Anode	Electrolyte	Average Loading (mg cm^−2^)	Current Density (mA g^−1^)	Charge/Discharge Capacity (mAh g^−1^)	ICE	Ref.
CPOP	1 M NaPF_6_ in EC:DMC (1:1 *v*:*v*)	3~4	30	300.6/339.3	88.6%	[40]
LPHC	1 M NaClO_4_ in TEGDME	-	50	202/220	92%	[41]
HC	1 M NaPF_6_ in DEGDME	1.5	20	275.4/287.5	85.9%	[42]
3DAC	1 M NaPF_6_ in EC:DMC (1:1 *v*:*v*)	2	30	280/373	75%	[37]
C/MCT	1 M NaPF_6_ in DME	1.4	20	311.9/416.9	74.8%	[43]
LDHC	1 M NaPF_6_ in DEGDME	-	100	303/407	74.4%	[44]
LSW-HC	1 M NaClO_4_ in EC:DMC (1:1 *v*:*v*)	2	25	284/364	79.1	[45]
HAHC	1 M NaPF_6_ in DME	0.8–1.2	50	293/490	59.7%	[46]
HC-HO	1 M NaSO_3_CF_3_ in DEGDME	3~4	15	314.4/340.6	92.3%	This work

Note: carbonized pre-oxidation pitch (CPOP), lithium-pretreated HC (LPHC), three-dimensional amorphous carbon (3DAC), lignite-derived hard carbon (C/MCT), hard carbon from lignin (LDHC), washing lignin sulphonate hard carbon (LSW-HC), hard carbon from sulfamethazine (HAHC).

## Data Availability

The original contributions presented in this study are included in the article/Supplementary Material. Further inquiries can be directed to the corresponding author.

## Supplementary Materials

The following supporting information can be downloaded at: https://www.mdpi.com/article/10.3390/mi16070771/s1, Figure S1: BET; Figure S2: Plateau and slope capacity; Figure S3: Rate performance of HC and HC-HO; Figure S4: Discharge/charge curves of HC-HO; Figure S5: Discharge/charge curves of HC; Figure S6: Performance comparison; Figure S7: NFM//HC-HO full cell; Figure S8: The EIS of pristine HC and HC-HO anodes; Figure S9: The EIS of HC and HC-HO anodes after 1 cycle; Figure S10: XPS; Table S1: The EIS of pristine HC and HC-HO anodes; Table S2: The EIS of HC and HC-HO anodes after 1 cycle; Table S3: The EIS of HC and HC-HO anodes after 200 cycles.
