# Supplementary material for: Surface Porousization of Hard Carbon Anode Materials for Sodium-Ion Batteries
Source: Micromachines (Basel). 2025 Jun 30;16(7):771. doi: 10.3390/mi16070771 (PMC12300324; doi:10.3390/mi16070771)
Supplement: Supplementary file 1 [file micromachines-16-00771-s001.zip › micromachines-3702116-supplementary.pdf]

Supplementary Materials

Surface Porousization of Hard Carbon Anode Materials for Sodium-Ion Batteries

Qianhui Huang [1], Shunzhang You [2] and Chenghao Yang [2,*]

[1] China Southern Power Grid Technology Co., Ltd., Guangzhou 510080, China; keelhuang1116@gmail.com
[2] Guangzhou Key Laboratory for Surface Chemistry of Energy Materials, New Energy Research Institute, School of Environment and Energy, South China University of Technology, Guangzhou 510006, China; 202210189203@mail.scut.edu.cn
* Correspondence: esyangc@scut.edu.cn

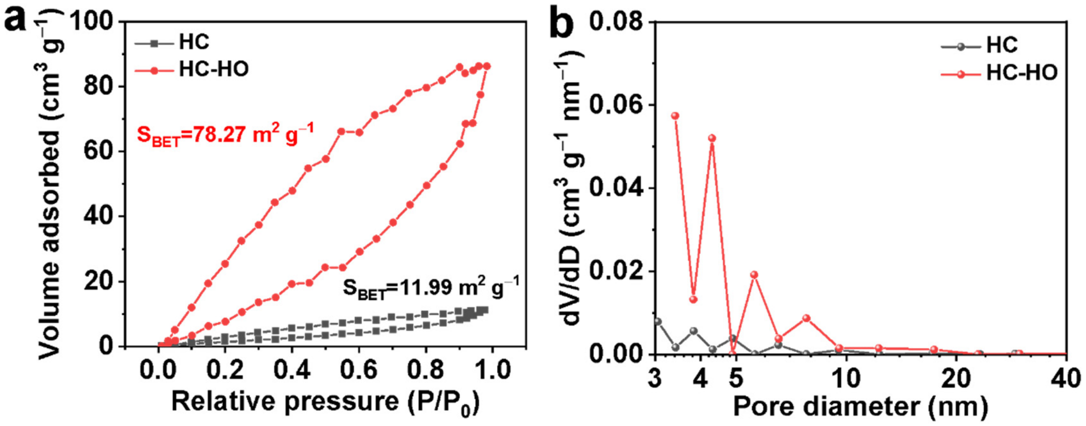

Figure S1. N_2 adsorption/desorption isotherm (a) and corresponding pore distribution (b) of HC and HC-HO.

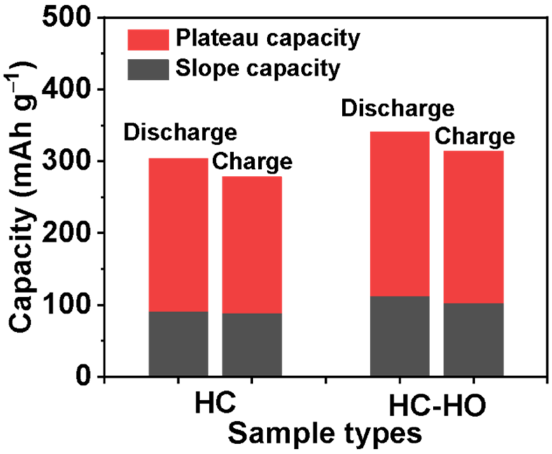

Figure S2. The plateau and slope capacity for first cycle at 0.05 C of HC and HC-HO.

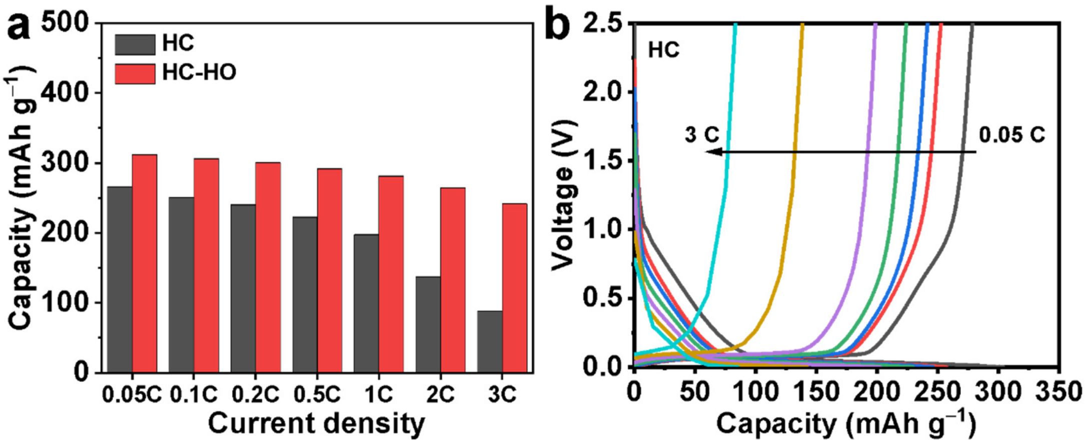

Figure S3. Rate performance of HC and HC-HO (a). Discharge/charge curves of HC from 0.05 to 3 C (b).

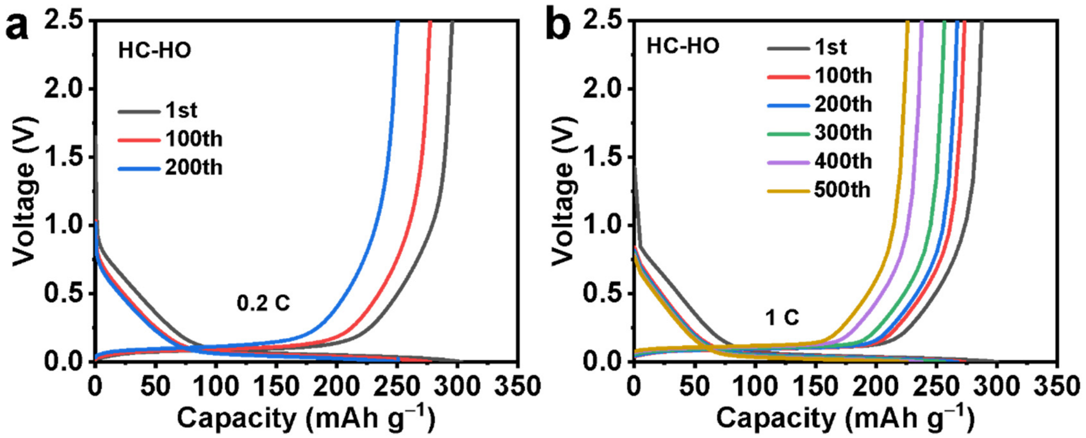

Figure S4. Discharge/charge curves of HC-HO at 0.2 C (a) and 1.0 C (b).

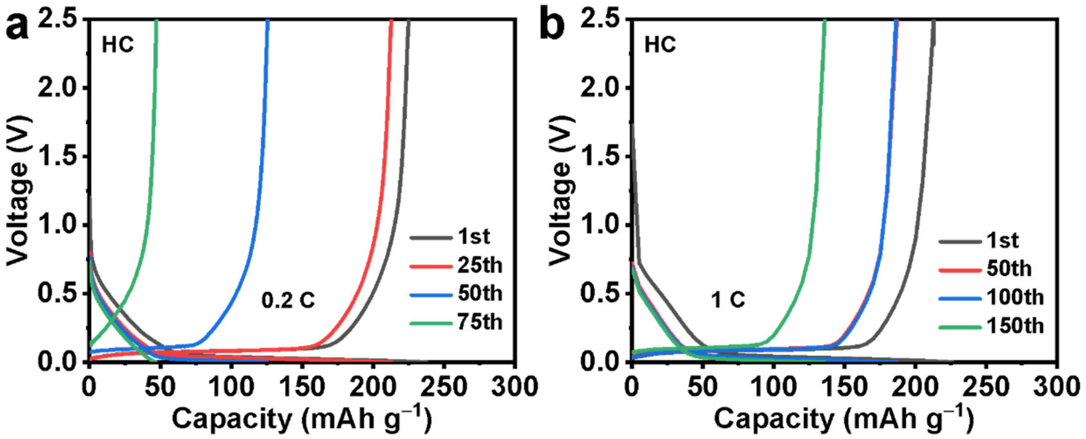

Figure S5. Discharge/charge curves of HC at 0.2 C (a) and 1.0 C (b).

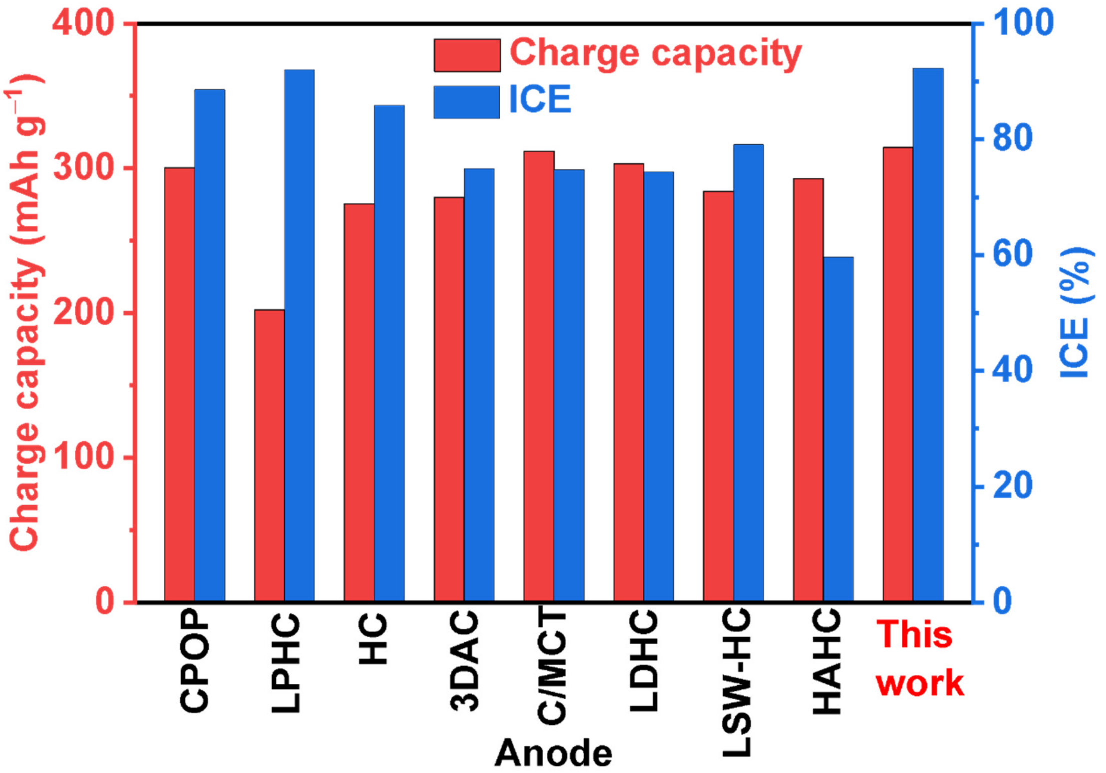

Figure S6. Comparisons of ICE and initial charge capacity between the HC-HO and HC anodes as reported elsewhere.

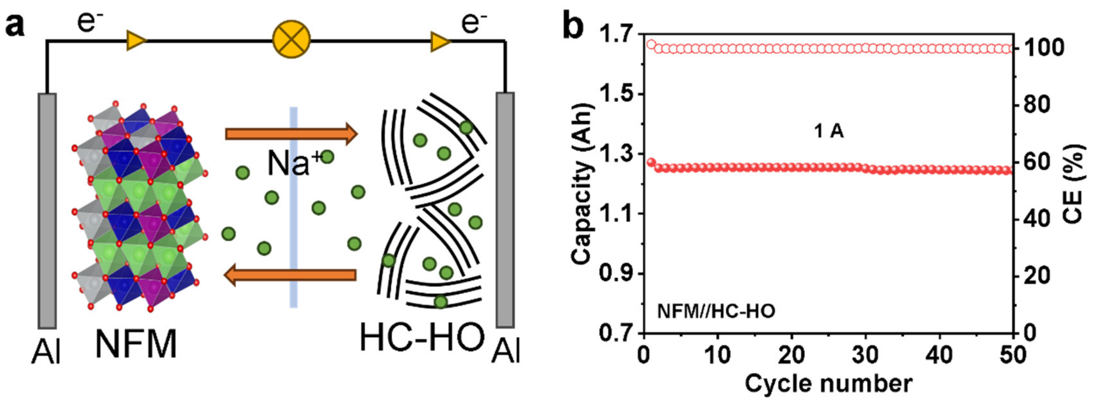

Figure S7. Schematic of the NFM//HC-HO full cell (a). Cycling performance of the full cell at 1.0 A (b).

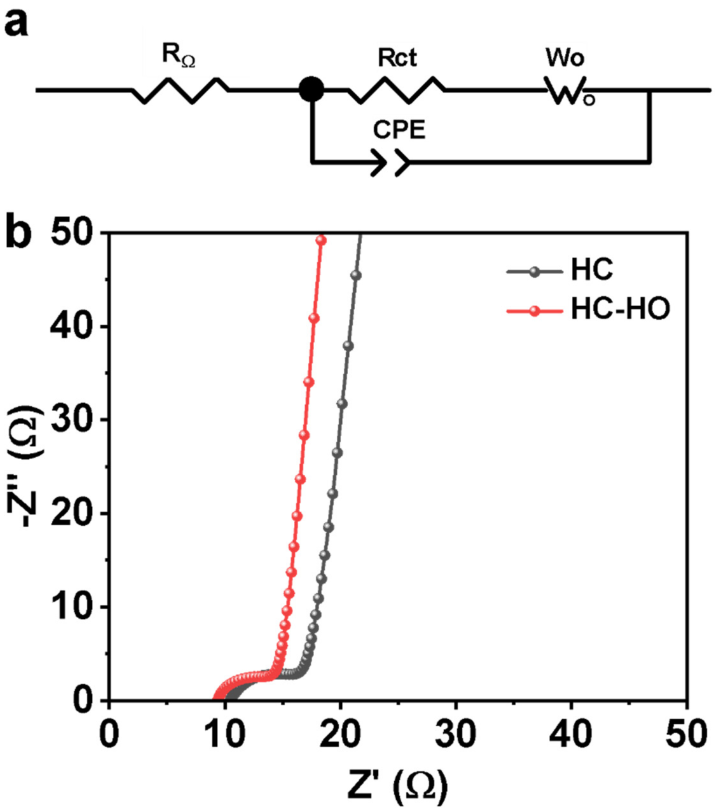

Figure S8. The equivalent circuit diagram (a) and EIS (b) of pristine HC and HC-HO anodes.

Table S1. Comparisons of EIS for pristine HC and HC-HO electrode.

Samples	R_{Ω}	R_{ct}
HC	10.48 Ω	8.844 Ω
HC-HO	9.397 Ω	7.226 Ω

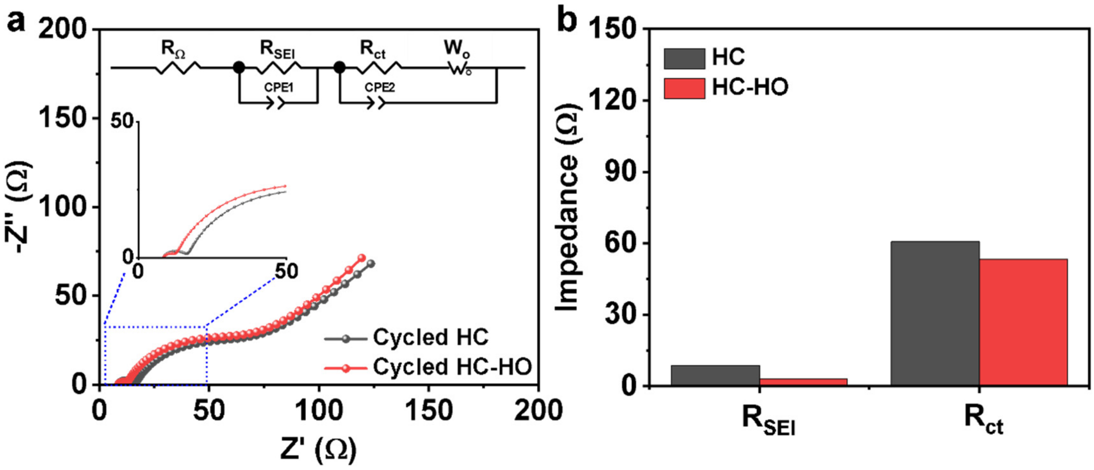

Figure S9. The EIS spectra (a) and the corresponding R_{SEI} and R_{ct} (b) of HC and HC-HO anode after 1 cycle at 0.2 C. The insets of (a) present high-magnification graph and equivalent circuit.

Table S2. Comparisons of EIS for HC and HC-HO anodes after cycled at 0.2 C for 1 cycle.

Samples	R_{Ω}	R_{SEI}	R_{ct}
HC	8.51 Ω	8.706 Ω	60.65 Ω
HC-HO	8.162 Ω	3.057 Ω	53.29 Ω

Table S3. Comparisons of EIS for HC and HC-HO anodes after cycled at 0.2 C for 200 cycles.

Samples	R_{Ω}	R_{SEI}	R_{ct}
HC	8.797 Ω	29.11 Ω	131.3 Ω
HC-HO	8.319 Ω	4.227 Ω	100.5 Ω

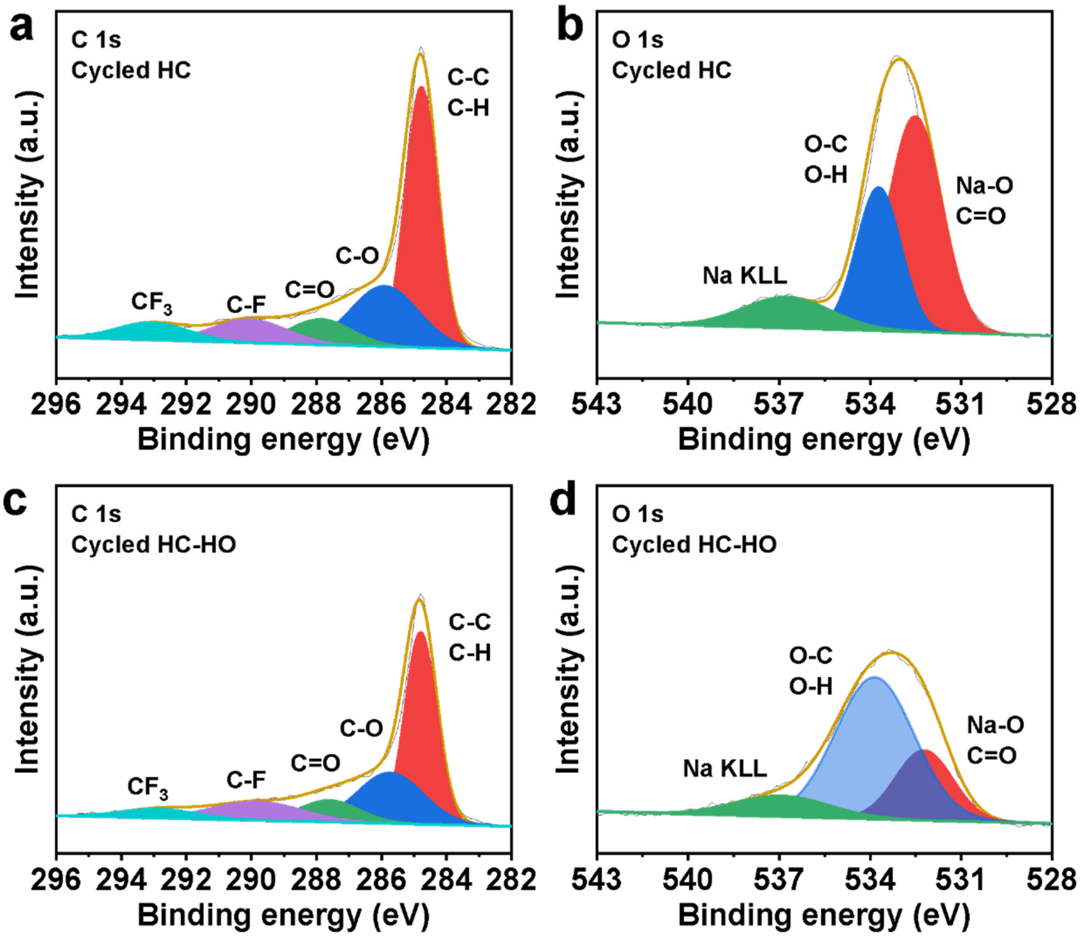

Figure S10. High-resolution XPS profiles of the cycled HC (a, b) and HC-HO (c, d) in the C 1 s and O 1s regions.
